# Literary Note

**Published:** 1908-08

**Authors:** 


### LITERARY NOTE.

Lea & Febiger, Publishers, Philadelphia, make announcement of a new edition of Gray's Anatomy. A thorough revision has been in progress for about two years and is so near completion that publication will be made during August.

Gray's Anatomy has maintained such a lead in its own field since its original publication fifty years ago that it has won the distinction of being the most important work in all medical literature. Hundreds of thousands of copies have started students at the beginning of their course in medicine, have been kept always at hand, and have been carried to their offices after graduation for guidance in the basic facts of medicine and surgery. Such an announcement as a new edition of "Gray" is therefore of primary importance to everyone concerned with medicine, whatever be his stage or station in medical life.

